# Chloroform in Labor

**Published:** 1875-02

**Authors:** 


Influence of Chloroform in Labor on the Fœtus.—Dr. Sweifel (*Berl. Klin. Woch.—Med. Reporter*) states that he has by chemical tests detected considerable chloroform in the placenta from a woman to whom the anæsthetic had been given only fifteen minutes. The child's urine also contained chloroform. He thinks that, from his clinical observations and these facts, the influence of anæsthetics upon the fœtus in utero is greater than is ordinarily supposed.—*Detroit Review of Medicine and Pharmacy.*

